# How negative life events affect emotional eating in Chinese adolescents: moderated mediation model

**DOI:** 10.1192/bjo.2025.63

**Published:** 2025-06-27

**Authors:** Rong Tan, Tao Huang, Yiru Li, Yuhe Zhang, Xijin Li, Xuanxuan Lin, Zhenjiang Liao, Qiuping Huang

**Affiliations:** Department of Psychology, School of Humanities and Management, Hunan University of Chinese Medicine, Changsha, Hunan, China; Mental Health Center, Dongguan Jiarong Foreign Language School, Dongguan, Guangdong, China; Mental Health Center, Jiangmen Preschool Education College, Jiangmen, Guangdong, China; School of Psychology, Fujian Normal University, Fuzhou, China; School of Education, Huazhong University of Science and Technology, Wuhan, China; Department of Psychiatry, National Clinical Research Center for Mental Disorders, The Second Xiangya Hospital of Central South University, Changsha, Hunan, China

**Keywords:** Negative life events, self-control, perceived social support, emotional eating

## Abstract

**Background:**

Emotional eating, the tendency to eat in response to negative emotions, is rising among adolescents and linked to obesity and mental health issues. While negative life events contribute to emotional eating, the roles of self-control and social support remain unclear.

**Aims:**

This study examined the relationship between negative life events and emotional eating in adolescents, testing self-control as a mediator and perceived social support as a moderator.

**Method:**

A sample of 740 Chinese high school students (aged 14–18) completed validated measures of negative life events, self-control, perceived social support, and emotional eating. Data were analyzed using SPSS 25.0 (IBM Corp., Armonk, New York, USA)and PROCESS macro for mediation/moderation effects.

**Results:**

Negative life events predicted higher emotional eating (*β* = 0.11, *p* < 0.01), while lower self-control mediated this relationship (*β* = −0.15, *p* < 0.001). Perceived social support moderated the association (*β* = −0.09, *p* < 0.05), weakening it among adolescents with stronger support.

**Conclusions:**

Negative life events increase emotional eating, but self-control and social support play key roles. Interventions targeting these factors may reduce emotional eating and improve adolescent well-being.

Emotional eating is regarded as a coping mechanism^1^ employed by individuals when facing stress or negative emotions, manifested by seeking solace in food to alleviate emotional discomfort. A multitude of researchers have empirically evidenced a substantial and escalating prevalence trend of emotional eating behaviours among children and adolescents. For example, one study claimed that more than 27% of girls aged 12–18 showed a gradual increase in emotional eating behaviours throughout adolescence.^2^ This phenomenon is intricately linked to the management and treatment trajectory of conditions such as obesity, thus accentuating its status as a pertinent societal concern deserving attention. Previous research has often associated socioeconomic status and gender with such eating disorders,^3^ indicating that emotional eating is more prevalent among individuals with lower socioeconomic status^4^ and within female populations.^5^ The observation that women display more severe patterns than men has led researchers to consider the influence of societal expectations on women’s behaviour.^5^ Intriguingly, Burke et al’s study^6^ contradicted this notion, revealing that the assumption that eating disorders are confined to lower socioeconomic groups is a misconception stemming from enduring biases; in reality, these disorders are present across all socioeconomic levels. In addition, other studies^5^ have corroborated that annual household income does not directly impact emotional eating behaviour; rather, socioeconomic status influences such behaviour under conditions like chronic stress. This suggests that risk factors for disordered eating related to individual socioeconomic status are complex and cumulative.^7^ Nevertheless, a consistent finding is that emotional eating correlates with negative self-perceptions concerning one’s appearance, health, weight and body shape,^8^ and even correlates with lower life satisfaction.^9^ Moreover, emotional eating has been identified as a potential risk factor for individuals’ mental well-being.^10^ For instance, emotional eating has been specifically linked to depression, suggesting potentially harmful implications for the mental health of adolescents.^11^ Recent research suggests that emotional eating ought to be acknowledged as a prevalent mental health concern among adolescents, with negative daily life experiences emerging as a significant predictor for this behaviour.^12^ This sentiment is echoed by a consensus among other scholars in the field.^13^

Negative life events are an ensemble of stimuli consisting of risk factors for various forms of psychopathology.^14^ These events encompass a wide array of stressors, such as traumatic experiences, chronic adversity, interpersonal conflicts and significant losses, all of which can profoundly impact an individual’s psychological well-being.^15^ The accumulation of negative life events has been associated with an increased vulnerability to mental health disorders, including anxiety, depression, post-traumatic stress disorder and substance misuse.^16^ Suffering represents a threatened emotional state after negative events that elicits adaptive physiological, cognitive and behavioural responses.^17^ Experiencing suffering caused by a negative life event can plunge an individual into this distressing emotional state, disrupting the equilibrium of their emotions and heightening the susceptibility to emotional disturbances. Empirical evidence supports the manifestation of such precarious scenarios, indicating that distress in individuals may influence their eating behaviours, thereby establishing a robust correlation between adaptive responses and emotional eating disorders, particularly prevalent among adolescents.^18^ A study focusing on emotional eating patterns among African American adolescent boys underscored this shift in attention, revealing that perceptions of discriminatory pressures from the environment could diminish eating restrictions and redirect focus towards the current stressor, potentially contributing to the development of obesity.^19^ Concurrently, negative life events can lead to emotional eating, thus triggering obesity issues.^20^

In addition, there is physiological evidence supporting the influence of negative life events on emotional eating. Prolonged stress, exemplified by the experiences of women during the COVID-19 pandemic, triggers a cascade of physiological alterations, notably heightened cortisol secretion, which can foster increased hunger.^21^ Chronic stress further has the potential to disrupt the normal functioning of the hypothalamic–pituitary–adrenal (HPA) axis, leading to aberrant neuroendocrine patterns characterised by heightened appetite and emotional reactivity.^3^

The significance of studying emotional eating and its problematic attributes in adolescents cannot be overstated, especially considering the constrained resources available to them and the self-evident impact of life events in such scenarios. Therefore, it becomes imperative to delve deeper into the correlation between negative life events and emotional eating in adolescents. Accordingly, this study is designed to explore how life stress events influence emotional eating among Chinese adolescents. Drawing from the existing literature, our initial hypothesis posits that negative life events are likely to positively correlate with the intensity of emotional eating in adolescents (H1).

Although research acknowledges a relationship between stressful life events and emotional eating, it is important to recognise that not everyone who faces stressful events will engage in emotional eating. From the perspective of the theory of conservation of resources, possessing adequate psychological resources equips individuals with the resilience required to cope effectively with such stressors.^22^ When examining how individuals adapt to negative life events, a critical consideration arises: what resources are available to those affected by such events? Understanding the availability and role of these resources is fundamental in clarifying why emotional eating varies among individuals who have experienced similar negative events. This inquiry is not only crucial for identifying the triggers for emotional eating but also in developing effective interventions and treatments. Among these resources, self-control stands out as a significant psychological asset that enables individuals to manage their responses and behaviours effectively.^23^ It is believed to play a pivotal role in how individuals handle the impact of negative life events and their propensity towards emotional eating.

Self-control is the ability of an individual to override or influence his or her own psychological, behavioural and physiological processes, that is, to carry out, regulate and control internal responses.^24^The diathesis-stress theory hypothesises that a person’s genetic predisposition interacts with environmental stresses as a shaping factor in the development of behavioural problems.^25^ Adolescence represents a crucial phase in both physical and mental development, during which individuals are particularly vulnerable. Stimulating events from the external environment, coupled with deficiencies in self-regulation and self-control, can elevate the risk of psychological and behavioural issues in this immature group.^26^ Research exploring the dynamics between stressful life events and self-control consistently shows a trend: higher levels of stressful life events correlate with diminished self-control.^27^ Moreover, self-control is inversely related to negative emotions such as anxiety and depression, as well as to problematic eating behaviours. Elevated self-control levels in individuals tend to foster better management of poor eating behaviours.^28^ High-pressure situations can impair cognitive control over eating, promoting overeating; individuals with higher self-control are less likely to resort to maladaptive eating behaviours.^27^ Consequently, negative life events and self-control are likely significant influences on emotional eating behaviours in adolescents. Encountering negative life events may weaken an individual’s self-control, potentially leading to an increase in maladaptive behaviours, such as overeating. Drawing from these findings, the current study posits a second hypothesis: self-control mediates the relationship between negative life events and emotional eating (H2).

Individual stress is difficult to alleviate without external support,^22^ making it crucial to identify buffering factors that assist individuals in overcoming the challenges posed by negative life events. Social support, particularly perceived social support, is fundamental in this context. It embodies the belief that assistance will be available from others when needed, reinforcing an individual’s ability to cope with adverse circumstances.^29^ Research indicates that individuals with limited social support tend to endure more negative emotions and experiences when faced with adverse life events, such as chronic illness, compared to those with ample social support who experience fewer negative outcomes under similar conditions, often resulting in greater emotional well-being.^30^ According to the theory of conservation of resources, individuals confronted with stressful events strive to mobilise resources to ameliorate their circumstances and counterbalance persistent challenges, which helps mitigate the adverse effects of stress. The process of losing or gaining resources plays a critical role in determining the nature of the stress response.^31^

Adolescents who are unable to mobilise resources effectively in response to negative life events are likely to experience elevated levels of psychological stress. Conversely, perceived support from others can significantly aid in coping with such stress. Research underscores the importance of peer group support in reducing psychological distress among students and lowering the incidence of emotional and behavioural problems.^32^ Moreover, a deficiency in social support is recognised as a significant risk factor for mental health issues among adolescents.^33^ Studies focusing on adolescent demographics confirm that social support can buffer the negative impacts of stress, thereby enhancing emotional health and well-being.^34^ While existing research has indicated the moderating role of social support in adolescent mental health, its specific moderating influence on the relationship between negative life events and emotional eating in adolescents remains underexplored. Therefore, drawing from the theory of conservation of resources and supporting empirical evidence, we propose our third research hypothesis: to investigate the moderating role of social support in the relationship between negative life events and emotional eating among adolescents (H3).

In summary, this study employs the theoretical frameworks of emotional eating, the relationship model between self-control and emotional eating and the theory of conservation of resources to construct a moderated mediation model (refer to Fig. 1). This model is designed to elucidate the impact of negative life events on adolescent emotional eating. By investigating this model, the study aims to reveal the underlying mechanisms through which negative life events influence adolescents’ emotional eating behaviours. The anticipated findings are expected to furnish empirical evidence that will inform targeted interventions and protection strategies.

**Fig. 1:**
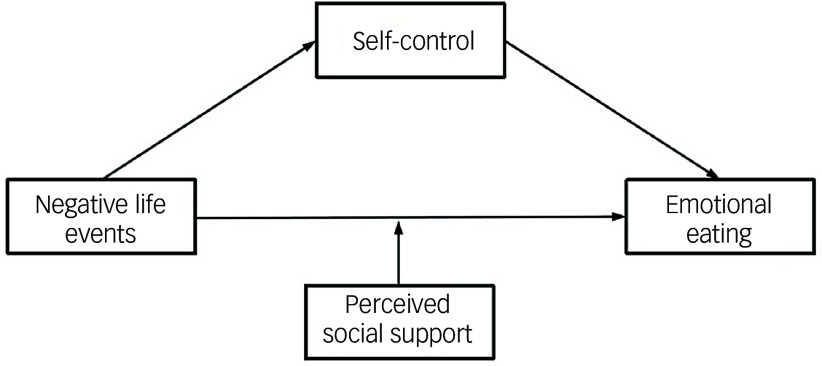
Hypothetical model.

## Method

### Participants

Using a cluster sampling method, a total of 740 senior high school students from Fujian provinces of China were initially recruited from 1 December 2023 to 1 January 2024. Before the questionnaire survey began, the researcher provided a detailed introduction to the study’s objectives and procedures. Participants were asked to carefully complete each question after providing written informed consent. Since the participants were minors, the classroom teacher also obtained oral informed consent from their parents via telephone, and the recordings were made and kept by researchers. All participants could withdraw at any time during the process of completing the questionnaire. This test lasted approximately 15 min. Incomplete responses and irregular responses were excluded. Subsequently, 722 valid data-sets were collated for analysis (mean age 15.56, s.d. age 1.17), with an age range of 14–18 years, achieving a validity rate of 97.57%.

Table 1 displays the details of all participants’ demographic information.

**Table 1:** Demographic information

Demographic information	Participants (*n*)	%
Gender		
Male	367	50.83
Female	355	49.17
Grade		
Senior one	477	66.07
Senior two	89	12.33
Senior three	156	21.61
Household registration		
Urban areas	640	88.64
Rural areas	82	11.36
Number of children in family		
One	454	62.88
More than one	268	37.12

### Measures

#### Negative life events

Negative life events were assessed by the Adolescent Self Rating Life Events Check List.^35^ This questionnaire comprises 27 items, with each item rated on a 6-point Likert scale, where ‘0’ means the event did not happen. If the event has occurred, it is rated on a scale of 1–5 (‘1’ for unaffected and ‘5’ for extremely heavily affected) based on the impact of the event (e.g. ‘Failed or unsatisfactory examination results’). Higher scores correspond to more negative life events experienced and more severe negative life events. In the current study, the Adolescent Self Rating Life Events Check List demonstrated strong internal consistency, with a Cronbach’s *α* of 0.87.

#### Self-control

The Dual-Mode of Self-Control Scale was utilised to gauge perception of self-control in adolescents.^36^ This questionnaire comprises 21 items (e.g. ‘I’m an impulsive man’), each measured using a 5-point scoring method (‘1’ indicating strongly inconsistent, ‘5’ indicating strongly consistent). A higher cumulative score indicates more pronounced self-control. The scale has demonstrated suitability for use with Chinese adolescents and exhibited good reliability in the current study (Cronbach’s *α* = 0.85).

#### Perceived social support

The assessment of perceived social support employed ‘The Perceived Social Support Scale’ (revised), as validated by Chen et al.^37^ This scale contains 12 items (e.g. ‘I am able to get emotional help and support from my family when needed’) and used a Likert 7-point scoring method, ranging from 1 (completely disagree) to 7 (completely agree). A higher total score indicates a higher degree of perceived social support. In this study, the Cronbach’s *α* of the Perceived Social Support Scale was 0.89.

#### Emotional eating

The 13-item emotional eating subscale of the Dutch Eating Behaviour Questionnaire was used for assessment of restrained, emotional and external eating behaviour. This subscale has been adapted and validated for use in the Chinese population^38^ to assess high school students’ emotional eating behaviours (e.g. ‘Do you crave food when you are provoked?’). Each item rated on a 5-point Likert scale (‘1’ indicating never and ‘5’ indicating very often). Higher total scores indicate more severe emotionally induced eating behaviours. The scale has demonstrated suitability for use with Chinese adolescents^39^ and exhibited good reliability in the current study (Cronbach’s *α* = 0.96).

### Statistical analyses

SPSS version 25.0 software for Windows (IBM Corp., Armonk, New York, USA) and the SPSS PROCESS macro program^40^ were employed to process all data, involving the following several steps. First, Harman’s single-factor analysis was utilised to examine potential common method bias. Subsequently, descriptive statistics and correlation analyses were conducted for each of the primary variables. Finally, the PROCESS macro (specifically Model 5) was utilised to evaluate the chained mediation model using 5000 bootstrap samples and 95% confidence intervals. The result is statistically significant if the lower and upper limits of the 95% confidence interval do not include ‘0’ (an index and test of linear moderated mediation).

### Ethics statement

The authors assert that all procedures contributing to this work comply with the ethical standards of the relevant national and institutional committees on human experimentation and with the Helsinki Declaration of 1975, as revised in 2013. This study was approved by the Ethics Committee of Fujian Normal University (code number: SCNU-PSY-2023-12-010). All participants were thoroughly informed about the objectives of this investigation and provided their signed informed consent. In addition, under the researcher’s supervision, the classroom teacher obtained oral informed consent from their parents via telephone, with recordings of these consents preserved for our records. All participants could withdraw at any time during the process of completing the questionnaire.

## Results

### Common method bias

Harman’s single-factor test was employed to examine potential common method bias.^41^ The analysis revealed 16 factors with eigenvalues above 1, and the variance explained by the first common factor was 14.26%, which is below the empirical threshold of 40%.^42^ Therefore, there is no serious common method bias in this study.

### Descriptive statistics and correlation analysis of the main variable

Descriptive statistics (means and standard deviations) and correlations for the main study variables are presented in Table 2. Specifically, self-control was significantly and negatively associated with emotional eating (*r* = –0.17, *p* < 0.01), while negative life events were significantly positively correlated with emotional eating (*r* = 0.15, *p* < 0.01). Furthermore, negative life events were significantly negatively correlated with self-control (*r* = –0.26, *p* < 0.01) and perceived social support (*r* = –0.25, *p* < 0.01). There was a significant positive correlation between core perceived social support and self-control (*r* = 0.26, *p* < 0.01).

**Table 2:** Results of descriptive statistics and correlation analysis

Variables	Mean	s.d.	1	2	3	4	5
1. Gender	–	–					
2. Negative life events	45.25	12.37	−0.033				
3. Self-control	37.83	13.62	−0.01	−0.26^**^			
4. Perceived social support	51.62	11.08	0.17	−0.25^**^	0.26^**^		
5. Emotional eating	5.33	4.06	−0.17^**^	0.15^**^	−0.17^**^	0.06	

*N* = 722; gender is a dummy variable, male 1, female 0. ^*^ *p* < 0.05, ^**^ *p* < 0.01, ^***^ *p* < 0.001.

Given that gender exhibited significant correlations with certain primary variables in the study, it was introduced as a control variable in the subsequent model tests to mitigate their potential influence on the results.

### Testing the mediating role of self-control

The three variables, namely negative life events (independent variable), emotional eating (dependent variable) and self-control (mediating variable), were standardised and the SPSS PROCESS macro procedure (model 4) was used to test the mediating effect of self-control. The results of the theoretical model and the bootstrap test results are shown in Table 3. After controlling for gender, negative life events significantly positively predicted emotional eating (*β* = 0.11, *p* < 0.01), indicating that the direct effect is significant. Furthermore, the mediating effect of self-control is established. Negative life events significantly negatively predicted self-control (*β* = –0.26, *p* < 0.001), and self-control had a significant effect on emotional eating (*β* = –0.15, *p* < 0.001), suggesting an indirect effect.

**Table 3:** Mediating effect and 95% CI estimate

Path	Model	Indirect effect estimation	CI 95%	
Total effect		0.15	0.08	0.22
Indirect effect	NLE–self-control–emotional eating	0.04	0.02	0.06

NLE, negative life events.

Therefore, these findings show that self-control partially mediates the relationship between negative life events and emotional eating. The mediating effect of self-control was estimated at 0.04, accounting for 26.67% of the total effect. The bootstrap 95% confidence interval for the mediating effect was [0.02, 0.06].

### Testing the moderated effect of perceived social support

Model 5 was used to test for the moderating role of perceived social support. The results indicate that the interaction term between negative life events and perceived social support significantly and negatively predicted emotional eating (*β* = –0.09, *p* < 0.05). In other words, perceived social support had a negative moderating effect on the relationship between negative life events and emotional eating.

**Fig. 2:**
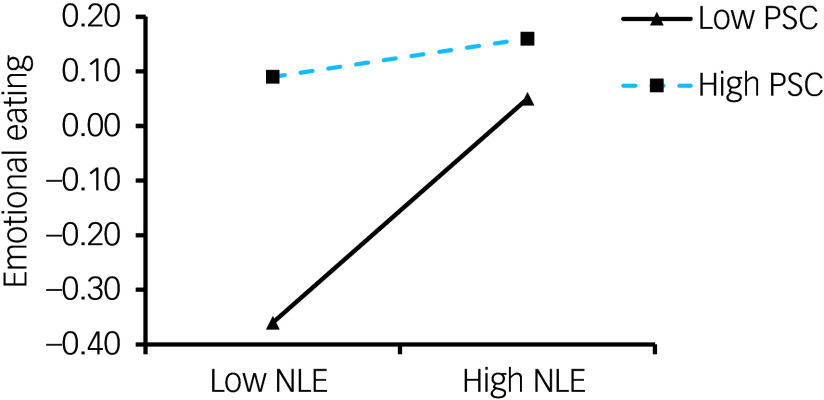
The moderating role of perceived social support (PSC) in the relationship between negative life events (NLE) and emotional eating.

**Fig. 3:**
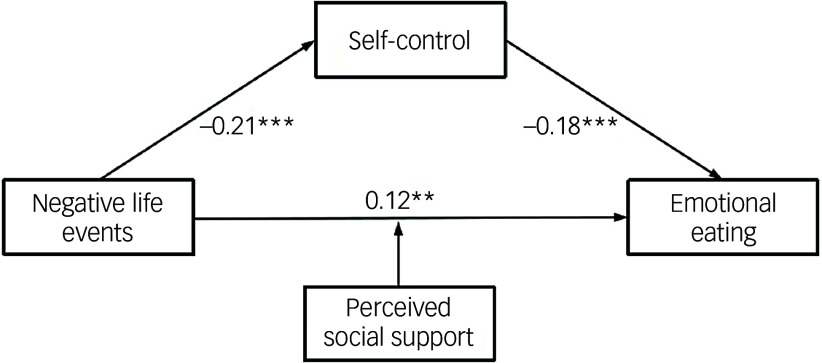
Moderated mediating effects of perceived social support and self-control. **p < 0.01, ***p < 0.001.

The direct effect values and 95% confidence intervals for negative life events on emotional eating were calculated at three levels of perceived social support scores: one standard deviation below the mean, the mean and one standard deviation above the mean. These values are presented in Table 4. Further simple slope analysis revealed compelling results. For high school students with low perceived social support (–s.d.), there was a significant trend indicating that as negative life events increased, emotional eating also increased (Bsimple 0.21, s.e. = 0.05, *p* < 0.001). This means that for every standard deviation increase in negative life events, there was a corresponding rise of 0.21 standard deviations in emotional eating. However, among high school students with high perceived social support (+s.d.), an increase in negative life events did not significantly predict a rise in emotional eating (Bsimple 0.04, s.e. = 0.06, *p* > 0.05). Notably, the prediction of emotional eating was weaker with high perceived social support compared to low perceived social support (see Fig. 2). This demonstrates the moderated mediating role played by perceived social support and self-control (refer to Fig. 3).

**Table 4:** Direct effects at different levels of perceived social support

Perceived social support	Intermediary effect	Bootstrap s.d.	Bootstrap low limit	Bootstrap upper limit
Mean – s.d.	0.21^***^	0.05	0.11	0.30
Mean	0.12^**^	0.04	0.05	0.20
Mean + s.d.	0.04	0.06	−0.07	0.15

**p < 0.01, ***p < 0.001.

## Discussion

This study explored the association between negative life events and emotional eating in adolescents, scrutinising the underlying mechanisms. The analysis revealed that negative life events, self-control, perceived social support and emotional eating in adolescents are components of a moderated mediation model. There is a noteworthy direct relationship between negative life events and emotional eating in adolescents, alongside an indirect influence exerted through self-control. Furthermore, perceived social support moderates the extent of this relationship, with more pronounced effects observed when levels of perceived social support are low. These findings clarify the pivotal roles that self-control and perceived social support play within the dynamics between negative life events and emotional eating in adolescents. Insights from this study underscore the importance of bolstering social support to alleviate emotional eating and foster positive, robust psychological growth in adolescents.

### The relationship between negative life events and emotional eating in adolescents

The results of the present study suggest that negative life events positively predicted emotional eating behaviour in adolescents, supporting the findings of previous studies in which negative life events were viewed as necessary prerequisites and drivers of the development of eating disorders.^43^ The stress-integration model further provides support for this,^16^ with emotional eating being an emotional coping mechanism employed to seek solace in times of stress.^1^ The trigger for emotional eating may be the individual’s urgent need to relieve stress after experiencing a negative life event, seeking a sense of compensation through binge eating, which subsequently exacerbates emotional eating. Individuals prone to emotional eating often experience significant emotional repercussions, display heightened impulsivity, and may underestimate their behaviours. Typically, they gravitate towards highly palatable, energy-dense foods, a shift that can contribute to the development of obesity and associated health complications.^13^

### The mediating role of self-control

The present study found that negative life events indirectly influence adolescents’ emotional eating behaviour through self-control. First, the level of self-control decreased with the exacerbation of negative life events, consistent with previous findings. Adolescents exposed to negative life events develop internalising psychological symptoms.^44^ Negative life events may disrupt individuals’ thoughts and alter their mood, impairing cognitive control over behaviour.^45^ Second, we found that self-control negatively predicts emotional eating, aligning with findings in obese patients,^46^ and enhancing self-control improves adolescents’ emotional eating behaviour. Zhu et al^28^ identify poor self-control as a critical factor contributing to emotional eating. Adolescents, in particular, are susceptible to diminished self-control following negative life events, which complicates their ability to regulate and curb overeating behaviours. This reduction in self-control leads to more pronounced issues with emotional eating and negatively affects both their physical and mental health. The mediational analysis reveals that self-control acts as a conduit through which external stressors, such as negative life events, erode an individual’s internal capacities (e.g. self-control), thereby influencing their eating behaviours. Consequently, interventions aimed at enhancing self-control among adolescents can help reduce the effects of negative life events and promote better mental health outcomes.

### The moderating role of perceived social support

Perceived social support has a moderating effect between negative life events and adolescents’ emotional eating, with moderation occurring in the main effect pathway. When the level of collateral social support is high, emotional eating behaviours caused by suffering negative life events are lower for adolescents. This suggests that adolescents’ level of perceived social support can help reduce the occurrence of emotional eating behaviours. Hobfoll’s theory of conservation of Resources emphasises that individual stress cannot be alleviated without external social support, and the results of this study support this theory.^22^ Furthermore, previous research has shown that perceived social support is not only a separate risk factor for adolescent mental health but also moderates the association between life events and mental illness,^47^ aligning with the present study’s findings. Indeed, our results indicate that perceived social support acts as a protective factor in reducing emotional eating among adolescents. This is consistent with the buffering model,^48^ which posits that social support can shield individuals exposed to life events from potential pathogenic factors, a view confirmed by numerous empirical studies.^49^ However, previous studies^50^ have found that adolescence is inherently a socially risky stage, with more psychological crises and challenges, making the role of adolescents’ social support systems even more crucial. This highlights the essential role of peer and family influences as protective factors against emotional eating in adolescence. Peer relationships, with their strong emotional and social support, profoundly shape adolescents’ food preferences and eating behaviours.^51^ Social cognitive theory suggests that positive peer environments – such as those in schools and communities promoting healthy habits – encourage adolescents to adopt healthier eating patterns by modelling friends’ behaviours and shared health beliefs.^52^ Family dynamics are equally crucial. Findings from other studies show that a supportive family environment fosters resilience, helping adolescents build healthier coping mechanisms for managing stress and reducing the risk of emotional eating. Besides, another important area of focus is community support. In recent years, community-based youth mentoring (CBM) programmes have gained traction in high-income countries like the USA, the UK, New Zealand, Canada and Ireland.^53^ These programmes, though varied in approach, commonly emphasise early intervention, family involvement and environmental sustainability.^53^ For instance, Ireland identifies at-risk youth early, teaching core competencies and building resilience.^54^ The USA adopts a multi-modal strategy to address youth needs holistically, while Canada integrates local cultural elements to fill gaps in youth mental health services.^55^ This underscores the need for society, families and schools to provide more resources for comprehensive adolescent support, helping to mitigate stress from negative life events, reduce behaviours like emotional eating and improve overall mental health.

Therefore, targeted measures should be implemented to help adolescents reduce impulsive maladaptive coping behaviours, such as overeating, and enhance their mental health. Schools and families should establish systematic communication channels and platforms to minimise the impact of negative life events on adolescents, reduce resource depletion and alleviate sources of stress. In addition, for adolescents who have already experienced negative life events, obtaining real and effective perceived social support is crucial. Insufficient support can impair the protective effect of perceived social support in buffering stress. Previous reviews of empirical studies on adolescent social support^56^ suggest that adolescents particularly need various types of social support for their physical and mental health development, indicating that the actual level of support received by adolescents may be inadequate. Thus, adolescents experiencing negative life events who develop behavioural problems like overeating are likely under-resourced, which can trigger further adverse consequences, such as a tendency to choose high-calorie foods, and induce additional psychological and health issues.

### Limitations

This study has several limitations. First, while the use of self-reported surveys provided valuable insights, the exclusive reliance on this method may have introduced reporting bias. Future studies should implement a multi-method approach incorporating physiological measurements such as cortisol levels, skin conductance, electroencephalography (EEG) and blood glucose monitoring to provide objective indicators of stress and eating behaviours.^57^ Second, as a cross-sectional study, it is challenging to determine causal relationships between variables and their effects on behaviour. We recommend future longitudinal studies that track participants over key developmental transitions (e.g. from junior to senior high school), include at least three measurement points over a 2-year period to capture change patterns and use growth curve modelling to analyse individual trajectories of emotional eating. Third, although the research methodology was robust, this study lacked direct engagement with participants. Future research should incorporate semi-structured interviews with adolescents to understand their lived experiences and focus groups to explore group dynamics and peer influences. In addition, the demographic variables in this study were somewhat limited; thus, future research should consider including factors such as socioeconomic status indicators (family income, parental education, occupation), parenting styles, family dynamics and school environment characteristics. Finally, while this study focused on the impact of external factors, such as perceived social support and negative life events, on adolescents’ emotional eating, it highlighted the importance of real and effective social support for adolescents’ healthy development. However, it did not offer a systematic and effective intervention programme for addressing adolescents’ behavioural problems. Future research should develop and test culturally adapted intervention protocols and conduct randomised controlled trials of prevention programmes.

In summary, this study explored the underlying mechanisms of negative life events and adolescents’ emotional eating behaviours from both theoretical and empirical perspectives. The findings clarified that exposure to negative life events indirectly affects adolescents’ emotional eating behaviours through the mediating variable of self-control. In addition, it was determined that perceived social support can moderate the relationship between negative life events and adolescents’ emotional eating behaviours. These results have practical significance in helping to effectively alleviate and reduce emotional eating and other behavioural problems in adolescents.

This paper validates the importance of social support, particularly within the unique context of Chinese adolescents. To enhance understanding, it is crucial to elaborate on how cultural factors influence social support and its impact on adolescent behaviour and emotional coping, especially compared to other cultural groups. Chinese culture values collectivism and family interdependence, influencing adolescent support systems. A notable example is intergenerational parenting, where grandparents play a significant role in child-rearing, especially in urban dual-income families.^58^ While this arrangement can ease parental pressures, it may also lead to the transmission of negative parenting styles, such as hostility and aggression, affecting children’s emotional well-being and extending into adulthood.^51^ For rural and migrant youth, limited resources pose additional challenges. Positive early experiences, such as family support and community engagement, are especially valuable in these populations, helping foster mental health and resilience.^59^ In contrast to Western individualistic cultures, where peer and institutional support are emphasised, Chinese adolescents rely more on family and community networks. This reliance can sometimes amplify stress from familial expectations, highlighting the importance of culturally tailored intervention models to address unique needs across different populations.

In summary, a localised, multilevel and multi-modal intervention system grounded in the Chinese cultural context and integrating school, family and community support is essential. Although the Chinese government mandates mental health services in schools, there remains a lack of a systematic intervention model, underscoring the need for a dedicated fund to implement these initiatives. At the school level, large-scale stress counselling workshops, adolescent stress management courses and group counselling sessions should be implemented.^60^ At the family level, national policies should emphasise family education’s importance, complemented by family education courses and parent–child workshops at the micro level. At the community level, community resources should be leveraged to create accessible, effective intervention pathways for at-risk adolescents, including timely support and family education guidance.^61^ In conclusion, future strategies should involve the collaborative participation of school teachers, family caregivers and community social workers to foster a comprehensive support network for youth.

## Data Availability

The data-sets used and/or analysed during the current study are available from the corresponding author on reasonable request.
